# Mechanisms That Regulate Peripheral Immune Responses to Control Organ-Specific Autoimmunity

**DOI:** 10.1155/2011/294968

**Published:** 2011-04-28

**Authors:** Gerard F. Hoyne

**Affiliations:** School of Health Sciences, University of Notre Dame Australia, 19 Mouat Street, Fremantle, WA 6959, Australia

## Abstract

The immune system must balance the need to maintain a diverse repertoire of lymphocytes to be able to fight infection with the need to maintain tolerance to self-proteins. The immune system places strict regulation over the ability of T cells to produce the major T cell growth factor interleukin 2 as this cytokine can influence a variety of immune outcomes. T cells require the delivery of two signals, one through the antigen receptor and a second through the costimulatory receptor CD28. The immune system uses a variety of E3 ubiquitin ligases to target signaling proteins that function downstream of the TCR and CD28 receptors. Mutations in these E3 ligases can lead to a breakdown in immune tolerance and development of autoimmunity. This paper will examine the role of a range of E3 ubiquitin ligases and signaling pathways that influence the development of T-cell effector responses and the development of organ-specific autoimmune diseases such as type 1 diabetes.

## 1. Introduction

The immune system has evolved to protect the body from infectious pathogens through both innate and adaptive immune responses. The adaptive immune response is built upon a diverse repertoire of antigen-specific T and B lymphocytes that learn to distinguish between self- from non-self- antigens during their differentiation in the thymus and bone marrow, respectively. Central tolerance is established in the thymus by the elimination of autoreactive thymocytes that display a TCR with high affinity for self-peptide/MHC complexes [1]. Despite the relative efficiency of clonal deletion not all tissue-specific antigens are expressed in the thymus and thus a small proportion of autoreactive T cells can escape thymic deletion, complete their maturation and enter the peripheral circulation. The immune system has multiple checkpoints in place to limit the activation and expansion of these autoreactive cells in the periphery [2]. The last decade has led to an improved understanding of some of these checkpoints involved in peripheral regulation of the immune response. 

Autoimmunity arises following a failure in either central or peripheral tolerance mechanisms. In the periphery the immune system has a range of mechanisms available that control the fate of autoreactive T cells, including immune privilege, immune ignorance, activation-induced cell death, clonal anergy, and immune suppression-mediated by regulatory T (Treg) cells [3–5]. Autoimmune diseases are classified as organ-specific or systemic depending on the source of the autoantigens. Type 1 diabetes (T1D) is an example of an organ-specific autoimmune disease caused by the breakdown in tolerance in both CD4+ and CD8+ T cells and B cells that express antigen receptors specific for proteins derived from the islets of Langerhans in the pancreas. Some of the key target autoantigens in T1D include insulin, GAD65, and insulin adenoma 2 (IA2) protein. Systemic autoimmune diseases are typified by systemic lupus erythematosus (SLE) or lupus and are due to the generation of high-affinity anti-self-antibodies specific for ubiquitous cellular proteins or nucleic acids. 

Type 1 diabetes arises as a result of a breakdown of tolerance in islet reactive T cells. This leads to immunological destruction of the pancreatic beta cells and loss of insulin secretion that is mediated by CD4+ and CD8+ T cells [6]. Patients can produce anti-insulin antibodies indicating that T1D reflects a generalized breakdown in immunological tolerance that allows islet-reactive CD4+ Th cells to provide help to autoreactive B cells. 

It was originally thought that clonal anergy and regulatory T cells were entirely distinct mechanisms that are used to control peripheral immune responses. However, studies in recent years have uncovered a remarkable overlap with the mechanisms of clonal anergy and also the generation of regulatory T cells in the periphery. In particular the discussion will examine the group of proteins known as ubiquitin ligases and how these proteins have emerged as an important class of negative regulators of the immune response not only in animals but also in humans. In addition I will examine how signaling through the AKT/mammalian target of rapamycin (mTOR) pathway has important roles in balancing the choice between immunity and suppression.

## 2. T-Cell Activation and Generation of Effector Responses

Naive T cells remain in a quiescent state as they circulate through secondary lymphoid tissues, the blood and lymph, and in the absence of an antigenic signal rely on survival signals transmitted via growth factor receptors (e.g., IL-7R, CD127) [7]. Importantly, quiescent cells are unable to secrete IL-2 because they recruit a number of different nuclear repressor proteins to the *Il2* gene locus (e.g., Ikaros, p50 NF-*κ*B dimers, Blimp1, Tob, and Smad proteins) that mediate epigenetic modification to repress the *Il2 *gene transcription [8–12]. 

T-cell activation is dependent on the delivery of two separate signals. Signal one is mediated through the TCR and signal two through the costimulatory receptor CD28 in response to binding of its ligands CD80/CD86 which leads to phosphorylation of a number of intracellular signaling pathways including phospholipase *γ*-1, protein kinase-*θ*, MAPK, JNK, PI3K, and I*κ*-B kinase (IKK) that leads to the recruitment of transcription factors (e.g., NFAT, AP-1, and NF-*κ*B) critical to the transcription of the *Il2* gene [13]. IL-2 is a multifunctional cytokine that plays a role in T-cell mitogenesis stimulating growth of activated T cells via paracrine and autocrine signalling through the IL-2 receptor (IL-2R) [14]. It can promote Th1 and Th2 cell differentiation; it is required for the generation of CD8+ T cell memory responses and plays a crucial role in the maintenance and homeostasis of Treg cells in the periphery. The delivery of a costimulatory signal during T cell activation leads to derepression of the *Il2* gene, and *Il2* mRNA transcripts become stabilized and translated into protein to drive T cell mitogenesis. Given the role that IL-2 has in coordinating the proliferative response of effector T cells, it is not surprising that the immune system has placed *Il2* gene transcription under tight regulation in an effort to restrict the inappropriate activation of this gene in naïve T cells *in vivo* and to limit the potential for a breakdown in immune tolerance and for autoimmunity. Therefore the secretion of IL-2 following T-cell activation represents an important checkpoint that can determine the outcome between immunity and tolerance [15].

## 3. Clonal Anergy 

Ligation of TCR on T cells in the absence of CD28 costimulation leads to the development of clonal anergy which is characterized by the failure to activate the MAPK, PI3K/AKT, and the IKK pathways and results in reduced activity of the nuclear factors AP-1 and NF-*κ*B and deficient IL-2 gene transcription, but there is elevated NFAT signalling in anergic cells [5]. The original studies performed on mouse Th1 cell clones *in vitro* showed that ligation of TCR in the absence of CD28 costimulation leads to a hyporesponsive state and a failure to secrete IL-2 leading to an abortive proliferative response [5]. It is also possible to induce anergy in naïve CD4+ and CD8+ T cells *in vivo* that is identified by a failure of these cells to secrete IL-2 and inflammatory cytokines, for example, TNF-*α* and IFN-*γ* upon restimulation with antigen. Around the same time, human CD4+ T cells were also shown to be subject to clonal anergy *in vitro* through cognate presentation of peptide between MHCII expressing T cells, (i.e., T-T presentation in the absence of APCs) [16]. The induction of anergy can also be induced by artificially elevating calcium levels which leads to activation of calcineurin and NFAT activity in the cells [17, 18]. Finally delivery of soluble antigens via the intravenous or mucosal routes is particularly conducive to tolerance induction that reflects many aspects of anergy [19–23].

Clonal anergy is an active process that requires new protein synthesis and is associated with an anergic gene expression profile that is characterized by increased expression of a number of E3 ubiquitin ligases including Cbl-b, Itch, and Grail [18]. Recent studies have also identified that the induction of anergy is associated with induction of numerous negative regulators of TCR signaling including *diacylglycerol kinase*, *caspase3*, *Traf6*, *Ikaros*, *Egr2*, *Egr3,* and *CREM* (cyclic AMP response element modulator) [18, 24–29]. Early studies showed that anergy was associated with defective Ras/MAPK activation and AP1 transactivation, but the causal relationship of Ras activation to anergy induction was difficult to prove [30, 31]. More recently gene expression profiling of anergic cells revealed that inhibitors of RasGRP1 were increased [18, 32] and diacylglycerol kinase (DGK) is required for the induction of T-cell anergy and is induced in response to NFAT. DGK functions by inhibiting Ras activation by diminishing the second messenger diacylglycerol that would activate RasGRP1 [32, 33].

## 4. Regulation of T-Cell Activation by E3 Ubiquitin Ligases

Multiple E3 ubiquitin ligases are required for regulation of lymphocyte development and activation and immune tolerance [34, 35]. Ubiquitination is a highly conserved process where proteins become tagged with ubiquitin, and this can target them for degradation by the proteasome. Ubiquitin is added by the sequential activity of three enzymes: E1 is an activating enzyme, E2 is a conjugating enzyme, and E3 is a ligase that attaches the ubiquitin moiety to the target protein. Proteins can either be subject to monoubiquitination or polyubiquitination (Table 1) [36, 37]. 

 Cbl-b and its close paralogue, c-Cbl, are RING (really interesting new gene) type E3 ligases that regulate cell surface expression of activated receptors with receptor tyrosine kinase activity (e.g., the EGF receptor) through endocytosis and targeting them for ubiquitin-mediated degradation. C-Cbl is required for TCR modulation in thymocytes, while the two proteins appear to function redundantly for promoting TCR downregulation in peripheral T cells. *Cblb* is induced during the induction of anergy [17, 18, 38], and its expression is regulated by the *early growth response genes* (*Egr*) *2* and *Egr3* [28]. However *Cblb* has a nonredundant role in the induction and maintenance of peripheral T-cell anergy. *Cblb*-deficient mice are susceptible to autoimmune diseases, and T cells display hyperproliferation in response to TCR signaling and produce IL-2 in the absence of a costimulatory signal. The Komeda diabetes prone (KDP) rat strain develops spontaneous islet-specific autoimmunity that is caused by a loss of function mutation in *Cblb,* but the emergence of spontaneous disease requires additional susceptibility factors, notably a diabetes-susceptible MHC haplotype [39]. The autoimmune regulator (Aire) gene is a transcription factor that controls the expression of tissue-specific self-antigens within the thymus. In particular it is crucial for clonal deletion of self-reactive lymphocytes [40–42]. Aire deficiency in both mice and humans can trigger spontaneous autoimmune disease such as type 1 diabetes with variable latency [40, 43], and it was recently revealed that *Cblb* acts as critical failsafe mechanism to help restrain autoreactive T cells in the periphery as a result of an AIRE deficiency [44]. Combined mutations in *Aire* and *Cblb* lead to a lethal autoimmunity with destruction of the exocrine pancreas and multiorgan inflammation within 5 weeks of life, whereas on their own neither gene mutation could trigger the lethal autoimmunity [44]. 

Similarly, we find that islet-specific autoimmunity is only caused by *Cblb* deficiency in mice when combined with a TCR transgene that makes high numbers of islet-reactive CD4 cells in their thymus and expresses the neoself-antigen hen egg lysozyme on pancreatic beta cells [45]. The *Cblb* deficiency does not affect negative selection of autoreactive T cells or the differentiation of natural Tregs that develop in the thymus [45]. However, the islet reactive T cells from *Clbb^−/−^ TCR x insHel* double transgenic mice proliferated strongly and produced cytokines following restimulation with self-antigen *in vitro* indicating a breakdown in T cell anergy compared to wild-type cells from *TCR x insHel* which were unresponsive to the Hel stimulation. We also observed that the Cblb deficiency reduced the formation of inducible Tregs in response to TGF-*β* signaling, and these cells were less suppressive compared to wild-type iTregs [45]. This will be discussed further below.

Itch is a HECT (homologous to the E6-associated protein carboxy terminus) type E3 ubiquitin ligase and is upregulated following induction of anergy [17, 46]. Itch has an N-terminal protein kinase C-related domain, four WW domains, and a C-terminal HECT domain. The Itchy mouse strain was identified as the naturally occurring non-agouti-lethal 18H mutation that leads to a loss of function mutation in the Itch gene [47]. Itch can promote the ubiquitination of multiple proteins including Bcl-10, JunB, Cbl-b, and PKC-*θ* [17, 48, 49]. Itch-deficient mice develop a spontaneous and lethal systemic proinflammatory disease consistent with a failure of peripheral tolerance. The disease is associated with an expansion of Th2-type T cells that trigger a chronic pulmonary interstitial inflammation with elevated levels of IgE antibodies [48]. PLC-*γ*1 and PKC-*θ* are induced by calcium/calcineurin signaling, and Itch targets both proteins for endosomal sorting and lysosomal degradation to attenuate TCR signaling [17]. Reduced levels of PLC-*γ*1 and PKC-*θ* shorten the longevity of the immunological synapse and thus reduce the interactions between T cells and APCs. In addition, Itch also targets Jun B for degradation which is required for the formation of the AP1 transcription factor [50].

 Neural precursor cell-expressed developmentally downregulated 4 (Nedd4) is a HECT E3 ligase that is also expressed in T cells and can target multiple proteins for ubiquitination and degradation including JunB, Cbl-b, c-Cbl, Notch, PTEN, PLC-*γ*1, and Bcl10 which indicates that there may be a functional overlap between Itch and Nedd4 in T cells. Using fetal liver (FL) chimeras, Yang et al. [51] showed that reconstitution of irradiated mice with Nedd4- deficient FL cells led to normal T-cell development but these cells proliferated poorly following stimulation *in vitro* and produced less cytokines and the recipient animals did not develop any sign of overt autoimmunity. The defective T-cell activation was independent of JunB but instead could be attributed to a failure to ubiquitinate and degrade Cblb. Therefore, Nedd4 is required to degrade Cbl-b in order for T-cell activation to proceed [51].

Ndfip1 was originally identified through its association with the HECT E3 ligase Nedd4 and was later found to be a negative regulator of Itch [52, 53]. *Ndfip1^−/−^* develops a severe inflammatory disease that manifests clinically similar to that observed in *Itch^−/−^* mice. The Ndfip1-deficient mice develop severe skin inflammation with weight loss, splenomegaly, hepatomegaly, and premature death. The similarity between the disease phenotypes of *Itch^−/−^* and *Ndfip1^−/−^* mice may relate to the target proteins regulated by Itch, for example, Jun B which is upregulated and can promote Th2 differentiation [48, 54]. However, other targets of Ndfip1 include p63, p73, and c-Flip, but it is not known if dysregulation of any of these targets was responsible for the disease phenotype.

Grail is a RING-type E3 ubiquitin ligase that was found to be induced following the induction of T-cell anergy in CD4+ T cells [55]. Ectopic expression of Grail in CD4+ T cells inhibits IL-2 production and proliferation following stimulation antigen pulsed APCs [55]. In addition over-expression of Grail in T cells can selectively inhibit RhoGTPase activity but does not affect Ras activation and MAPK signalling [56]. This suggests that Grail has a separate regulatory function in controlling TCR signaling that is independent of the *Cblb* E3 ligase. Grail-deficient mice were resistant to immune tolerance induction, and they were more susceptible to autoimmune diseases [57]. Similar to the *Cblb^−/−^* mice, naïve T cells from Grail-deficient animals display a hyperproliferative response and increased cytokine production in the absence of a costimulation. In addition, loss of Grail function Tregs displays reduced suppressive function. 

Traf6 is an E3 ligase with an N-terminal RING finger domain, and one of its targets includes the NF-*κ*B essential modifier (NEMO) which is a member of the I*κ*B kinase complex that functions downstream of TCR signalling. T-cell-specific deletion of Traf6 leads to a multiorgan inflammatory disease with splenomegaly and lymphadenopathy. The Traf6 deficient cells display Th2 polarization and the mice produce increased levels of IgG1, IgE, and IgM. In addition, the TraF6-deficient T cells hyperproliferate in response to anti-CD3 only in the absence of CD28 costimulation, and this was related to increased phosphorylation of PI3K p85 and AKT [26, 58].

Roquin (*Rc3h1*) is a newly described RING finger protein with E3 ligase activity. The Sanroque mouse strain was identified through an ENU mutagenesis screen for regulators of systemic autoimmunity. The *Rch*31^san/san^ mice carry a point mutation in the conserved Roq domain generating a hypomorphic allele that gives rise to a lethal spontaneous systemic lupus-like disease [59]. The *Rch*31^san/san^ mice have spontaneous germinal centre formation, increased numbers of T follicular helper cells (Tfh), increased serum levels of double stranded DNA antibodies, and elevated serum Ig levels. Tfh cells of the *Rch*31^san/san^ mice constitutively express high levels of inducible costimulator molecule (ICOS) which can promote spontaneous germinal centre responses [60]. Roquin regulates ICOS expression levels by binding to the 3′ untranslated region of the *Icos* mRNA, promoting its degradation [61]. In the sensitized *TCR x insHel* model whereby the HEL neoself-antigen is expressed on the pancreatic islets [62], the expression of a high frequency of islet reactive T cells expressing the HEL-specific TCR, these *Rch*31^san/san^ *TCR x insHel* mice have rapidly develop type 1 diabetes and generate anti-Hel islet-specific autoantibodies [59]. This is an unusual feature of autoimmunity in this animal model as *Cblb^−/−^ TCR x insHel* mice do not make anti-Hel islet autoantibodies despite developing type 1 diabetes at high frequency [45].

When the Roquin mutation was combined with the Aire deficiency in mice, it was predicted that this combination would lead to rapid autoimmune disease given the severity of systemic autoimmunity observed in the *Rch*31^san/san^ mice. However, the *Aire^−/−^ Rch*31^san/san^ mice displayed normal survival rates and remained overtly healthy with no clinical signs of autoimmunity for over 140 days. In contrast, *Aire^−/−^ Cblb^−/−^* mice died with a mean survival age of 25 d with severe weight loss, and surprisingly the immunological destruction was restricted to the exocrine tissue of the pancreas [44]. As a result, the mice did not become diabetic as the islet tissue was unaffected by the autoimmune response. These studies highlight that *Cblb* appears to have a unique role in restricting the activation of autoreactive T cells in the periphery that is especially crucial when Aire-mediated clonal deletion is compromised in the thymus. 

There has been some significant progress made in the mechanisms that control TCR signalling and activation in the peripheral immune system. These checkpoints are crucial to prevent *Il2* gene transcription and inappropriate activation of autoreactive T cells. The immune system has multiple ways of dampening the TCR signal that is controlled by multiple E3 ligases. The specificity of each of these ligases is crucial to control immune responses under different environmental conditions. The outcome of responses may be influenced by the range of cytokines or even different subsets of APCs present within different tissues.

## 5. *Cblb* and * Itch* in Regulatory T Cell Development

Regulatory T cells that express the forkhead/winged helix transcription factor Foxp3 play a critical role *in vivo* in suppressing immune responses. Mutations in Foxp3 in mice (scurfy) and humans with immunodysregulation, polyendocrinopathy, enteropathy, and X-linked syndrome (IPEX) both lead to overwhelming multiorgan autoimmunity, diabetes, and early death [63–65]. Numerous studies have shown that Foxp3 is a master regulator of Treg development and function [66–68]. Overexpression of Foxp3 in naïve T cells can convert them to a Treg phenotype that enables them to suppress the response of other T cells either *in vitro* or *in vivo * [66–68]. Foxp3 is a transcriptional repressor that is recruited to the *Il2* and *Ifn*γ** loci in T cells and modulates histone acetylation [69]. It is now apparent that there are two types of Tregs in the immune system. The natural Tregs (nTregs) are selected in the thymus during CD4+ T-cell differentiation; these cells represent ~5–10% of the peripheral CD4+ T cell pool and play crucial roles in regulating T-cell responses to self-antigens [4]. The second subset is known as inducible Tregs whereby naïve CD4+ CD25− T cells cultured in the presence of TGF-*β* (IL-10 and IL-2) can switch on Foxp3 and differentiate as a Treg cell [70–73]. These iTregs function in an equivalent manner in being able to suppress responses of other T cells whether* in vitro *or *in vivo*. The mechanism of suppression by Tregs is mediated by secretion of inhibitory cytokines (e.g., IL10, IL-35, or TGF-*β*) and/or cell-cell contact [4]. 

TGF-*β* has long been known to exert anti-inflammatory effects on the immune system. Deletion of TGF-*β* leads to abnormal T cell responses and rapid autoimmunity [72, 74–76]. It also plays an important role in Treg generation and maintenance [72, 73] and together with IL-2 is important for the thymic development of nTregs [77]. TGF-*β* is also a potent inhibitor of T-cell proliferative response. However, in recent years there has been a significant advance made in understanding the plasticity of CD4+ Th cell lineage commitment. The Th1-Th2 paradigm stood unchallenged for nearly 20 years but in the last 5 years several new Th cell subsets have been defined. Activation of naïve T cells in the presence of TGF-*β* + IL-6 can induce ROR*γτ* expression, and this promotes the differentiation of Th17 cells which play critical roles in bacterial immunity as well as being implicated in autoimmune diseases [78]. Investigating how TGF-*β* influences the differentiation of Th cell subsets with completely distinct effector functions (i.e., Treg (suppression) and Th17 cell autoimmunity) has been a major focus for immunologists. It is now clear that when T cells encounter TGF-*β* alone this can be sufficient to induce *Foxp3* expression and allow iTreg development to proceed [70–73]. In contrast, the presence of IL-6 antagonizes Foxp3 expression by a direct effect on AKT activity [79] (discussed further below). 

TGF-*β* signalling by the TGF-*β*IIR leads to the phosphorylation of Smad2/3 which forms a complex with Smad4 and this complex is translocated to the nucleus to regulate transcription of target genes [80, 81]. One of the targets is Smad7 which is an inhibitory Smad used to attenuate TGF-*β* signalling by competing with Smad 2/3 for binding to the receptor [80, 81]. The intron 1 of the *Foxp3* gene in T cells contains two NFATs and a Smad3 binding sites that are located upstream of the Stat5 and CREB binding sites [82]. Naïve CD4+ CD25− T cells from *Cblb^−/−^* and *Itch^−/−^* mice show poor induction of *Foxp3* expression, and the iTregs induced are functionally less suppressive in coculture experiments with wild-type naïve T cells [45, 46, 83, 84]. 

To understand the molecular basis of Itch regulation of Foxp3 expression in iTreg cells studies by Liu and colleagues identified that TGF-*β*-induced early gene product (TIEG1 or KLF10) is induced in response to TGF-*β* stimulation [84]. Itch can bind to and ubiquitinate TIEG1 leading to both mono- and polyubiquitinated forms. Itch and TIEG1 can bind to the of *Foxp3 *promoter leading to its transactivation. Forced expression of TIEG1 in wild-type T cells can induce robust *Foxp3* expression, and cell proliferation was inhibited in the presence of TGF-*β* [84]. In contrast, overexpression of TIEG1 in *Itch*-deficient T cells led to poor induction of *Foxp3,* and these cells were resistant to TGF-*β* inhibition *in vitro*. Furthermore, analysis of TIEG1*^−/−^* T cells demonstrated that they were also resistant to the inhibitory effects of TGF-**β* in vitro,* they failed to induce *Foxp3* expression in the presence of TGF-**β* in vitro, *and they were less effective in mediating suppression of growth of wild-type naïve T effector cells. Finally, replacing TIEG1 in TIEG1*^−/−^* T cells through retroviral transduction could restore *Foxp3* expression, and these cells could mediate immune suppression [84]. Taken together these results have identified an important pathway by which Itch and TIEG1 controls iTreg cell development by regulating Foxp3 expression. This is likely to have important implications in the control of autoimmune responses to tissue-specific antigens in the periphery.

## 6. Regulation of T-Cell Activation by Sensing the Environment

The AKT-mTOR pathway has emerged as a central checkpoint that controls the fate of multiple cell types in the immune system [85–87]. Signaling downstream of the TCR and growth factor receptors (e.g., IL-2R) leads to activation of PI3 kinase (PI3K), the serine threonine kinase AKT, and mTOR which in turn leads to increased activity of the cyclin-dependent kinase (e.g., CDK2) that allows cells to traverse the G1 cell cycle restriction check point to stimulate proliferation and avoid the induction of anergy. The AKT-mTOR pathway is highly conserved and is used by eukaryotic cells to integrate environmental cues such as nutrient and amino acid availability, senses energy stores, and directs cellular proliferation [88]. The AKT-mTOR pathway stimulates a switch in cellular metabolism from catabolic to anabolic pathways in activated T cells leading to an increase in glycolysis and the expression of nutrient transporters [88]. Recent studies have revealed that AKT/mTOR signaling is crucial for the survival and homeostasis of naïve and memory T cells, the differentiation of CD4+ Th cell lineages (e.g., Th1, Th2 Th17) [89], CD8+ T cell memory [90, 91], as well as the differentiation of Treg cells in the periphery [79] and lymphocyte trafficking [92]. AKT can in turn activate mTOR and induce the phosphorylation of the Foxo transcription factors (e.g., Foxo 1 and Foxo 3a) which leads to their nuclear export and subsequent degradation [87]. Treatment of T cells with the mTOR inhibitor rapamycin could block mTOR activity and induce T cell anergy even when T cells received a TCR and costimulatory signal [93]. The induction of anergy requires the complete inhibition of mTOR activity, and there is heightened activation of the calcineurin/NFAT pathway [15]. The AKT-mTOR pathway acts downstream of several energy sensing pathways in eukaryotic cells, that can modulate expression of genes that are involved in the maintenance of anergy, or indirectly mediates degradation of the gene products [85].

## 7. mTOR in Treg Development and Function

As discussed above, activation of naïve T cells in the presence of TGF-*β* readily induces expression of *Foxp3*. This induction requires proteins that normally block PI3K activity such as Cbl-b and PTEN [94, 95]. Likewise, constitutive AKT activity in T cells can inhibit Foxp3 expression [79]. Delgoffe et al. [89] recently showed that deletion of mTOR leads to increased expression of *Foxp3 *in T cells and that mTOR deficient CD4+ T cells are hypersensitive to TGF-*β* treatment and switch on *Foxp3*. The effect on *Foxp3* expression appears to be related to mTORC2 activity and not mTORC1. The Foxo transcription factors, Foxo1 and Foxo3a, are nuclear proteins that function downstream of AKT/mTOR signaling, and their activity is regulated by AKT-mediated phosphorylation that inactivates them and they are exported from the nucleus. One of the targets of the E3 ligase Cbl-b is the p85 subunit of PI3K. Active PI3K is required for activation of AKT and so in anergic cells, which express higher levels of Cbl-b, can inactivate PI3K signaling leading to inhibition of mTOR/AKT signalling. Two recent studies have identified that regulation of the Foxo1 and Foxo3a transcription factors is essential for TGF-*β*-mediated iTreg cell differentiation [83, 96]. 

In the absence of *Cblb* the Foxo1 and Foxo3 proteins become phosphorylated indicating heightened levels of PI3K-AKT signaling, and this leads to impaired expression of Foxp3 in *Cblb^−/−^* T cells when cultured in the presence of TGF-*β* [83, 96]. The loss of *Cblb* does not impact on nTreg differentiation in the thymus and does not influence cell numbers in the periphery. Ouyang et al. found that in the absence of both Foxo1 and Foxo3a T cells were unable to induce Foxp3 expression and when cultured in the presence of TGF-*β* did not affect nTreg differentiation. The iTregs from double knockout mice also showed increased expression of proinflammatory genes (e.g., IL-17 and IFN-*γ*) which are normally absent from iTreg cells. In addition, many of the Treg signature genes were also modulated in these cells. What is surprising is that the PI3K/AKT/mTOR/Foxo pathway appears to be more active in the iTreg cells and not the nTreg cells that develop in the thymus. From the recent studies it appears that AKT/mTOR signaling only affects the induction of Foxp3 expression and it does not have any influence on the maintenance of Foxp3 expression in iTreg cells.

## 8. Concluding Remarks

The development of T1D is caused by the breakdown of immune tolerance to islet-specific antigens in the pancreas. At present we do not understand how the breakdown in immune tolerance to islet-specific antigen occurs in most individuals. The last decade has seen some important developments in the identification of key genes that play an essential role in the regulation of peripheral immune responses as well as the further clarification of autoimmune checkpoints that try to limit organ-specific autoimmune diseases such as T1D. The recent discovery regarding the plasticity of CD4+ Th cell development *in vivo* has led to a greater insight into the process of disease pathology. Understanding the molecular regulation between inflammatory T cell subsets and Treg cells offers the potential for therapeutic intervention in diseases such as T1D.

Clearly the immune system places an important effort in controlling the activation of naïve T cells as it strives to prevent inappropriate T effector cell activation and differentiation. As highlighted in this paper the family of E3 ubiquitin ligases play a critical role in the immune system and dysregulation of any of one of these genes can have important implications in the development of autoimmunity by disrupting processes such as clonal anergy, Treg differentiation, and generation of autoantibody responses. The next decade should hopefully see even more exciting discoveries as the fruits of various genetic screens in both mice and humans should begin to uncover immune checkpoints that have not been previously identified and should help to provide a better understanding of the disease process of T1D in humans that may eventually lead to the “holy grail” where we can cure people of this debilitating and life-threatening disease. 

## Figures and Tables

**Table 1:** E3 Ligases that play an important role in preventing autoimmunity.

Name	References	Type of E3 ligase	Function
Cbl-b	[40–45]	RING type	Ubiquitinates PLC-*γ*1 and PKC-*θ* downstream of TCR signalling. Also binds to and ubiquitinates P85 subunit of PI3K to inhibit CD28-mediated triggering of PI3K. Critical role in T-cell anergy.
Itch	[46–50]	HECT type	Important role in Th cell differentiation. Binds to a number of substrates including Notch, c-Flip, Smad2, p63, and p73. Ubiquitinates Bcl-10, JunB, Cbl-b, and PKC-*θ* and targets them for degradation. Binds to and is regulated by the E3 ligase Ndfip1.
Nedd4	[51]	HECT type	Binds to and ubiquitinates a number of targets that targets them for degradation including JunB, Cbl-b, c-Cbl, Notch, PTEN, PLC-*γ*1, and Bcl-10.
Ndfip1	[52–54]	HECT type	Associates with Nedd4 and is a negative regulator of Itch. Other targets include p63, p73, and c-Flip.
Grail	[55–57]	RING type	Selectively inhibits RhoGTPase activity but does not affect the Ras/MAPK kinase pathway. Inhibits IL-2 production and T-cell proliferation.
Traf6	[26, 58]	RING type	Binds to and ubiquitinates NEMO targeting it for degradation. Leads to upregulation of NF-*κ*B signalling in response to TCR signaling.
Roquin	[59–61]	RING type	Binds to RNA-protein complexes and targets themfor degradation. Important role in posttranscriptional regulation of target gene expression. Targets include ICOS, CXCR5, PDCD1, CCL5, Il21, and CD100.
